# Providing Real-Time Wearable Feedback to Increase Hand Use after Stroke: A Randomized, Controlled Trial

**DOI:** 10.3390/s22186938

**Published:** 2022-09-14

**Authors:** Diogo Schwerz de Lucena, Justin B. Rowe, Shusuke Okita, Vicky Chan, Steven C. Cramer, David J. Reinkensmeyer

**Affiliations:** 1 AE Studio, Venice, CA 90291, USA; 2 CAPES Foundation, Ministry of Education of Brazil, Brasilia 70040-020, Brazil; 3 Flint Rehabilitation Devices, Irvine, CA 92614, USA; 4 Department of Mechanical and Aerospace Engineering, University of California Irvine, Irvine, CA 92697, USA; 5 Department of Anatomy and Neurobiology, University of California Irvine, Irvine, CA 92697, USA; 6 Rehabilitation Services, University of California Irvine, Irvine, CA 92697, USA; 7 Department of Neurology, UCLA, Los Angeles, CA 90095, USA

**Keywords:** wearable sensing, IMU, hand movement, dexterity, rehabilitation, stroke, feedback

## Abstract

After stroke, many people substantially reduce use of their impaired hand in daily life, even if they retain even a moderate level of functional hand ability. Here, we tested whether providing real-time, wearable feedback on the number of achieved hand movements, along with a daily goal, can help people increase hand use intensity. Twenty participants with chronic stroke wore the Manumeter, a novel magnetic wristwatch/ring system that counts finger and wrist movements. We randomized them to wear the device for three weeks with (feedback group) or without (control group) real-time hand count feedback and a daily goal. Participants in the control group used the device as a wristwatch, but it still counted hand movements. We found that the feedback group wore the Manumeter significantly longer (11.2 ± 1.3 h/day) compared to the control group (10.1 ± 1.1 h/day). The feedback group also significantly increased their hand counts over time (*p* = 0.012, slope = 9.0 hand counts/hour per day, which amounted to ~2000 additional counts per day by study end), while the control group did not (*p*-value = 0.059; slope = 4.87 hand counts/hour per day). There were no significant differences between groups in any clinical measures of hand movement ability that we measured before and after the feedback period, although several of these measures improved over time. Finally, we confirmed that the previously reported threshold relationship between hand functional capacity and daily use was stable over three weeks, even in the presence of feedback, and established the minimal detectable change for hand count intensity, which is about 30% of average daily intensity. These results suggest that disuse of the hand after stroke is temporarily modifiable with wearable feedback, but do not support that a 3-week intervention of wearable hand count feedback provides enduring therapeutic gains.

## 1. Introduction

More than seven million stroke survivors currently live in the United States [1]. Approximately 50% have long term upper extremity (UE) motor deficits [2,3]. Their movement impairment is typically more severe for one hand (which we will refer to as the “impaired” hand), causing them to favor the use of their other hand (the “unimpaired hand”, although it should be acknowledged that this hand is typically not without some motor deficits [4]). Disuse of the impaired hand further reduces its movement ability in a “vicious cycle” [5,6].

Wearable technologies have proven useful for increasing walking activity, and thereby, promoting health of people without disabilities [7,8]. Daily step count feedback, accompanied by goal setting, is an effective way to increase walking activity and thereby improve difficult to change health outcomes, such as body mass index and blood pressure [9]. Here, inspired by the success of pedometers, we sought to determine if daily “hand count” (instead of “step count”) feedback, accompanied by goal setting, could increase the hand activity of people in the chronic phase following a stroke.

Such a wearable feedback approach for the UE after stroke is not yet well studied (for review see [10]). Perhaps the most relevant study is a pilot study in chronic stroke that focused on testing the effect on chronic stroke participants (N = 8) of providing regular and detailed therapist coaching sessions based on measurements of UE movement quantity provided by a wrist accelerometer [11]. This strategy significantly increased participants’ perception of paretic UE activity, yet no improvements of actual activity as measured using the accelerometers was found. Another relevant study [12] designed a wristband that adjusted the frequency of delivery of a vibration prompt based on the activity level of the wearer. This study showed the feasibility of this “remind-to-move” approach: the wristbands were worn for 79% of the recommended time (between 8 a.m. and 8 p.m.). The participants (N = 33, 0 to 3 months after stroke) also showed a preference for hourly prompts and not more frequent prompts. However, while clinical outcome measures were acquired, no statistical comparisons were made with a control group in this pilot feasibility study.

**Figure 1:**
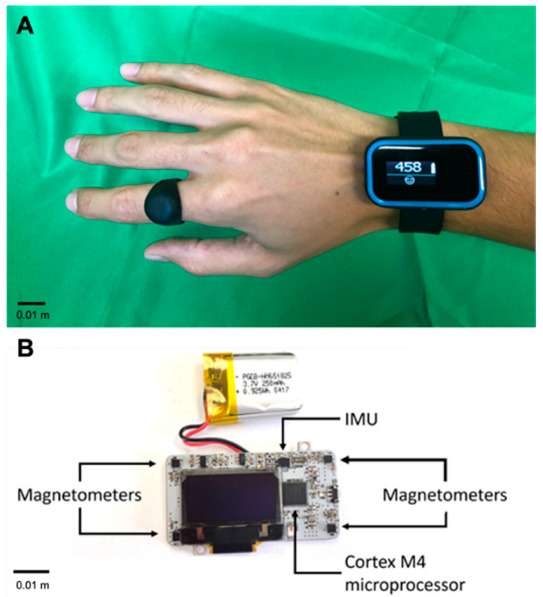
The Manumeter. The Manumeter with the companion magnetic ring (**A**), and the Manumeter board (**B**), with four magnetometers positioned at the corners of the board, an IMU with 6 degrees of freedom, and a non-ferromagnetic battery. All components are controlled by a Cortex M4 microcontroller and the display showing the number 458 is an OLED display.

Our desire to study real-time feedback of hand counts motivated us to develop a novel sensing approach. Currently, the most common way to measure UE activity outside the clinic is wrist accelerometry. Two potential limitations of this method are: (1) it may count non-functional arm movements; and (2) accelerometry “counts” are typically calculated in a way that summarizes activity over periods, which differs from how pedometers present discrete “step counts”. To address these issues, we developed the Manumeter [13,14,15,16], which is based on the principle of using changes in the magnetic field caused by movement of a magnetic ring to identify hand and wrist activity (Figure 1). We also developed and validated an algorithm (Hand Activity based on Nonlinear Detection (HAND)) that accurately counts discrete hand and wrist movements in a way that is analogous to step counts [16].

The primary goal of this study was to investigate the effect of providing real-time feedback on hand counts using the Manumeter and the HAND algorithm. We hypothesized that participants who received hand count feedback would increase their hand counts, and, consequently, increase the motor ability of their UE through a “virtuous cycle” [6].

## 2. Materials & Methods

### 2.1. The Manumeter

The Manumeter (Figure 1) is a jewelry-like device for monitoring wrist and finger movement. Four magnetometers mounted at the corners of a wristwatch-like device measure changes in the magnetic field produced by the movements of the magnetic ring worn on the ipsilateral index finger. Wrist accelerometry, which we used here to determine the orientation of the forearm with respect to gravity, can also be obtained using the included 6 DOF IMU. The Manumeter also has an OLED display that we used to provide real-time, visual feedback on amount of hand use. For more details on the design, construction, and initial testing of the Manumeter, see [13,14,15,16].

In this study, we measured “hand counts” using the previously validated HAND algorithm, where a single hand count could arise from a finger flexion/extension, wrist flexion/extension, or wrist radial/ulnar deviation movement. The HAND algorithm uses a thresholding approach based on magnetic field changes to identify discrete movements [16]. In the previous validation study, we evaluated the effects of hand size, movement types, and movement speed on hand counts. We found that the hand counts assigned using the HAND algorithm were strongly correlated with, but overestimated by ~20%, the actual number of hand movements. Overcounting is due, in part, to spontaneous, false positive counts, due to encountering stray magnetic fields in the environment. These false positive counts typically happen at a rate of about 100–200 counts/hour. The algorithm also tends to undercount smaller and slower movements [16].

### 2.2. Clinical Assessments

Several clinical assessments were performed by a blinded evaluator to evaluate inclusion/exclusion criteria, baseline function and impairment, and improvement over time. They include:

- Box and Blocks test (BBT): participants move 1-inch blocks from one side of the box to the other over a divider. The score is the number of blocks moved within the 1 min allotted for the test. For some of our analysis, we use the BBT ratio, which is the ratio between the participant’s BBT score using their impaired arm and the score using their unimpaired arm.
- Fugl-Meyer (Upper Extremity) scale (FMUE): consists of 33 tasks each scored from 0 to 2 points, with 2 corresponding to the successful execution of the task, 1 to partial execution, and 0 to minimal or no execution. A total of 66 points can be achieved.
- Action Research Arm Test (ARAT): participants attempt to perform 19 tasks from four groups, namely grasping, gripping, pinching, and gross arm movement. Each task requires transport of an object from starting to ending point, receiving a score of 0 to 3 points based on predefined performance criteria, for a total of 57 points.
- Motor Activity Log (MAL): asks participants to self-assess their performance in 28 daily activity tasks compared to before their stroke. They score, from 0 to 5 points, each task for two scales on amount of use (MAL-A) and how well (MAL-HW) for a total of 140 points in each of the scales.
- NIH Stroke Scale (NIH SS): a 15-item test assessing the severity of symptoms associated with a stroke for different skills, and not limited to motor impairment. Each item is scored on a 4-point scale for a total of 42 points, with higher scores meaning more impairment.
- Grasp and grip strength: a quantitative assessment of isometric grip strength using a standard hand dynamometer. The test is repeated three times for each hand.
- Mini-Mental State Exam (MMSE): a quantitative assessment of cognitive impairment composed of 11 questions with a maximum possible score of 30 points.
- Modified Ashworth Scale (MAS): measures resistance (tone) to passive movements for different muscles. The final score is an average across the assessed muscles with values spanning from 0 to 4, with 4 meaning the highest resistance.

### 2.3. Participants

We recruited 22 chronic stroke survivors for this parallel-group, assessor-blinded, randomized control trial. Participants in the study met the following criteria: (1) 18 to 80 years of age, (2) experienced one or more strokes at least six months previous, (3) Fugl-Meyer Upper Extremity Score < 60 (out of 66), (4) absence of substantial upper limb pain (<3 on the 10-point visual-analog pain scale), and (5) ability to understand the instructions to operate the device. Participants with implanted pacemakers were not allowed in the study for safety reasons concerning potential interactions with the magnetic ring. We limited the number of participants who scored zero on the Box and Blocks Test (BBT) to six at baseline.

Participants were recruited through our stroke survivor database, the outpatient clinics at U.C. Irvine, and regional hospitals and stroke support groups. All trials were performed at U.C. Irvine, and all participants provided informed consent according to a protocol approved by the local Institutional Review Board. The study was pre-registered on ClinicalTrials.gov (NCT03084705).

### 2.4. Sample Size

We based the power analysis on data from another study on a wearable hand training device, the MusicGlove [17], which was used at home by people with chronic stroke to increase their finger movement activity using a musical computer game. We found an effect size of 1.2 for within-subject improvement after three weeks. We expected the increase in activity due to feedback from the Manumeter to at least match the increased activity from MusicGlove training. Thus, 11 participants in each group gave us an 80% chance to demonstrate a difference between the feedback and control group with alpha = 0.05 (one-tailed), assuming a <10% dropout rate. Based on our previous work, we expected a low dropout rate.

### 2.5. Hand Count Goal Setting

**Figure 2:**
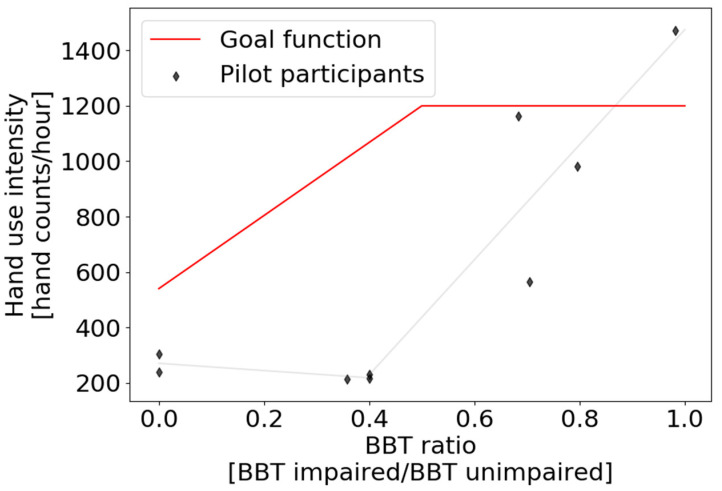
Hand count intensity goal function (red line) based on the BBT ratio between the impaired and unimpaired hands. The function was based on data acquired from pilot participants who wore the Manumeter during one day (diamonds). After the hand count intensity goal was defined, the daily goal was obtained by multiplying the intensity goal by the 10 h of expected daily wear time.

For each participant, we defined a personalized daily goal for hand counts, based on their BBT score as measured at the beginning of the study, using a new equation proposed in this study (Equation (1), Figure 2). We did not change the goal across the three-week intervention. Participants who exhibited at least 50% of the BBT score in their non-paretic hand were given a goal of achieving 1200 counts/hour for 10 h of Manumeter use per day (i.e., 12,000 counts). This number was comparable to the hand counts of young, unimpaired office workers, which we had measured in pilot testing using the Manumeter. For participants with a BBT score below 50% in the non-paretic hand, we defined a novel goal function that increased linearly from 540 to 1200 counts/hour.

(1) $$Intensity\;goal\;\left(BBT\;ratio\right)=\{\begin{array}{cc} 1320*BBT\;ratio+540,\; & BBT\;ratio<0.5\\ 1200,\; & BBT\;ratio\geq 0.5 \end{array}$$

Here, BBT ratio is the ratio of BBT score in the impaired to the unimpaired hand and the intensity goal is given in hand counts/hour.

We designed this novel goal function based on a pilot study with 9 chronic stroke survivors (6 males and 3 females) with an average (±SD) age of 67.9 ± 8.8 years and BBT score of 24.7 ± 20.3 blocks; five had a stroke affecting their dominant side. Participants wore the Manumeter for one day during daily activities for an average of 9.21 ± 1.75 h without receiving count feedback. Figure 2 shows their hand use intensity as a function of their BBT ratio (impaired/unimpaired hand). Note the apparently nonlinear relationship between their normal hand counts sampled for one day and their hand BBT ratio. Hand counts did not increase until the BBT ratio reached ~0.4. This relationship is consistent with the so-called “Threshold Hypothesis of Hand Use” [18], which we verified recently in a pilot study with the Manumeter [16]. This Threshold Hypothesis posits that persons with hemiparesis after stroke use their impaired hand rarely unless the functional capability of their impaired hand (as we measured in the clinic) exceeds a threshold. Overlaid on Figure 2 is the daily goal that the algorithm of Equation (1) would have generated for these participants. One can see that the assigned goal is particularly challenging for individuals who have moderate functional motor capacity (i.e., BBT ratio between 0.2 and 0.5), but are not using their hand for daily activities.

### 2.6. Study Design and Clinical Outcome Measures

The trial was composed of two baseline assessments (three to 10 days apart), a post-therapy examination after the three weeks of the hand training interventions, and a follow-up assessment three months after the end of the hand training interventions.

The primary clinical outcome was the BBT score measured three months after completion of the hand training period of the study. Secondary outcomes were the Fugl-Meyer (Upper Extremity) Scale (FMUE), Action Research Arm Test (ARAT), Motor Activity Log (MAL, including the How Well (MAL-HW) and Amount (MAL-A) subscores), and amount of upper extremity activity measured using the Manumeter. The BBT and FMUE were performed at the two baselines, post-therapy, and follow up. The remaining secondary outcomes and other relevant clinical tests were performed at the first baseline, post-training, and at the three-month follow-up.

The first baseline consisted of acquiring demographic information, stroke details, hand dominance, and clinical testing: BBT, FMUE, ARAT, MAL, NIH Stroke Scale (NIH SS), grasp and grip strength, Mini-Mental State Exam (MMSE), Modified Ashworth Scale, and Visual Analog of Pain scale. All the clinical data were collected by an experienced and blinded physical therapist. During clinical testing, participants wore Manumeters (with the screens turned off) on both wrists, and the magnetic ring on the index finger of the impaired hand (only during BBT scoring of the unimpaired hand was the magnetic ring switched to the index finger of the unimpaired hand). Before leaving the lab, participants were fitted with another Manumeter on their unimpaired ankle and asked to keep the Manumeter and ring on for the remainder of the day during their normal daily activities (other than showering, bathing, or swimming). Here, we use the data from the impaired hand Manumeter as the baseline to analyze the increase in the amount of hand use for each participant once they started the hand training intervention; we do not, in this paper, analyze the data from the other sensors they wore at baseline. The first baseline was performed primarily in the morning to allow more hours of baseline activity. Participants were asked to ship the Manumeters and the ring back using a prepaid shipping box.

At the second baseline assessment, performed three to 10 days after the first baseline, participants played a grip strength tracking game [19] and used the FINGER robot to measure finger proprioception [20]. The results of these two assessments will be presented in another paper. The BBT and FMUE were performed again. Participants then received a booklet of tabletop exercises tailored to them by a physical therapist and were instructed to practice the exercises for a total of three hours per week.

At the end of the second baseline, participants were randomized into feedback and control (i.e., no feedback) groups with a 1:1 ratio and balanced for BBT with separated randomizations for participants with impaired hand BBT > 0 and those with BBT = 0. At this stage, all participants were asked to wear a single Manumeter for the next three weeks on their paretic arm and the magnetic ring on the index finger of that hand, keeping the magnet facing upwards. They were also instructed to orient the screen such that the text was readable from their perspective. The Manumeter is water-resistant, but not waterproof, so participants were instructed to take the device off when showering, bathing, or swimming. Even though the Manumeter’s battery lasted for more than two full days of use, participants were asked to charge the device every night while sleeping. A binder with this information and instructions on how to charge the device was provided to each participant. Participants were instructed to remove the magnetic ring when cooking or when in proximity to sharp ferrous objects or MRI machines.

**Figure 3:**
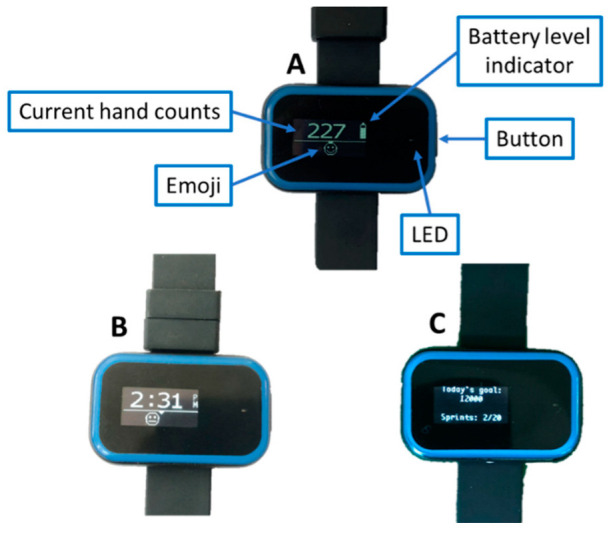
Manumeter screens shown to the participants in the feedback group. The counts screen (**A**) showed their current number of hand movement performed during the day, battery indicator, and the feedback emoji. The time screen (**B**) showed the current time as well as the emoji feedback. Participants in the control (i.e., no feedback) group were shown screen B without the emoji feedback. The goal screen (**C**) showed their daily goal of hand counts, number of sprints performed that day, and daily goal of sprints.

Participants randomized to the no-feedback group wore Manumeters that still recorded activity but showed only the time of day on the screen. Participants randomized to the feedback group were taught to read each of three available screens for the Manumeter: the counts screen, the goal screen, and the time screen (Figure 3). The counts screen showed their current number of hand movements (i.e., hand counts) performed during the day, battery indicator, and an emoji that gave feedback on their progress. The goal screen showed their daily goal of hand counts, number of “sprints” performed that day, and daily goal of sprints (for the definition of “sprints”, see the next section). The time screen showed the current time as well as the emoji feedback. To change screens, participants pushed the button on the side of the device. We instructed participants with BBT scores of 0 in the feedback group to increase hand activity by helping move the impaired hand with the unimpaired hand.

### 2.7. The Emoji Feedback and “Hand Sprints”

An emoji displayed on the watch screen was used in two different ways during the study. For the first three participants in the feedback group, the emoji represented their performance in the last 10 min towards the daily goal. We calculated their current hand count rate (counts/hour for the last 10 min) and compared it to the goal count rate—the count rate needed to get to their goal at 8 pm. The goal count rate was limited to two times their hourly goal. The emoji’s “happiness level” and left-right position on the screen depended on the goal ratio (ratio between the current count rate to the goal count rate). Goal ratios between 0 and 1 showed a frowning emoji to the left of the screen (with 0 being all the way to the left) and goal ratios greater than 1 displayed a smiley emoji to the right of the screen (with 2 being all the way to the right). We also programmed the LED on the Manumeter to blink at a rate inversely proportional to the goal ratio if the goal ratio was below 1; it did not blink when goal ratio was above 1. The LED only blinked if the user rotated their forearm to view the Manumeter screen, as gauged by the accelerometer. After the daily goal was achieved, the emoji and LED feedback were turned off.

We altered the nature of the emoji feedback after the first three participants experienced the initial emoji feedback scheme and provided feedback to us. The initial feedback scheme required us to estimate a time for the end of the day (we estimated 8 p.m.), but the actual time that participants stopped wearing the Manumeter varied substantially from day to day. Thus, the real-time goal count rate was often inaccurate. Second, participants did not like the blinking light for social reasons. Third, users suggested that the feedback should be more immediate and engaging. It was difficult for them to see an immediate cause–effect relationship between increasing hand use intensity and the emoji feedback, as the initial algorithm considered data over the last 10 min. Therefore, we removed the blinking LED and changed the emoji component of feedback to a “game-on-the-wrist” concept to make the Manumeter feedback more engaging.

Specifically, we implemented a “hand sprint” game for the remaining eight participants, which encouraged intense bursts of hand movement. In this game, participants had to get 30 hand counts in 30 s while their forearm was oriented such that they were viewing the Manumeter screen. We instructed them that they could achieve this by repeatedly opening and closing their hand for 30 s. As they did this, the Manumeter automatically that detected a “hand sprint” was in progress and moved the emoji in real-time from left to right and from frowny to smiley. When they “drove” the emoji all the way to the right (by achieving 30 hand counts in 30 s), a celebration was displayed with a motivating, fun message. Other motivating messages were shown during the game as the user achieved the 25%, 50%, and 75% milestones. The emoji was reset to the left of the screen and a frown if participants substantially changed the orientation of their arm (i.e., which was consistent with looking away from the screen). We asked them to perform 20 hand sprints per day. Note that the goal feedback screen stayed the same through the study for all 11 participants who received feedback; only the emoji feedback varied.

## 3. Data Analysis

We calculated change in hand use intensity using linear regression on average daily hand use intensity plotted versus day of use during the intervention part of the study. We also compared hand use intensity between groups during the baseline and follow-up assessment periods, when feedback was turned off, using two sample *t*-tests.

For the BBT and FMUE scores, which were the two assessments we measured at both baseline assessments, we averaged them to derive a baseline score. We used a linear mixed-effect model to test significance of time, group (intervention vs. control), and time and group interaction on all clinical outcomes. The model allowed random intercept and slope for each participant. All statistical analyses were performed in R, with the significance level set to 0.05.

## 4. Results

**Figure 4:**
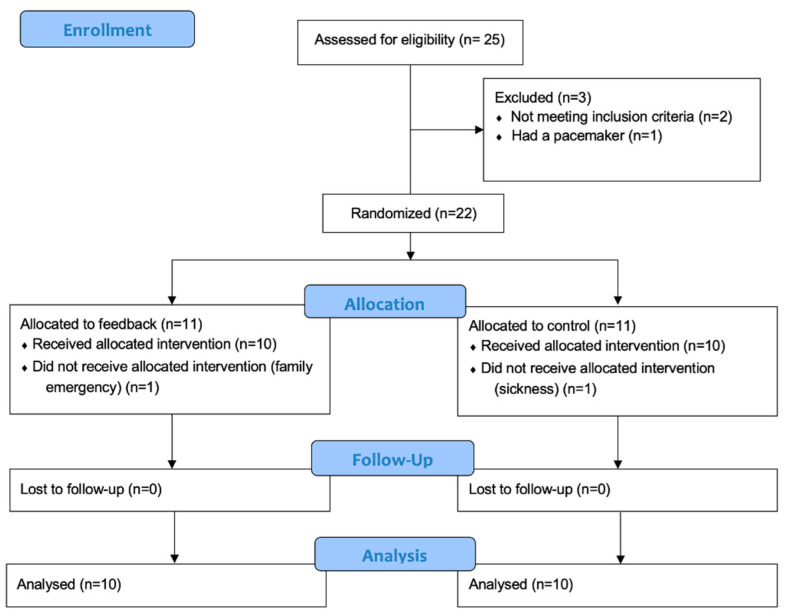
CONSORT flow diagram.

A total of 25 stroke survivors were screened for this study; 22 participants met the inclusion criteria and were successfully recruited. Half of them were randomized into the control group (no-feedback) and the other half into the feedback group. Two participants dropped out during the three weeks of intervention—one due to a family emergency and another due to sickness (Figure 4). Table 1 shows the demographic data and baseline clinical assessments. Some of the Manumeter data were lost during the intervention period:

- Total data loss (*n* = 2, feedback group): technical problems when downloading the data from the device
- Partial data loss (*n* = 2, one in each group): technical problems when downloading the data from the device
- Partial loss (*n* = 1, feedback group): device was stolen from car two weeks into the intervention. Device was promptly replaced
- Partial data removal (*n* = 1, feedback group): lost the magnetic ring. Ring was replaced after 6 days. Participant kept using the Manumeter without the ring during that period
- Total data removal (*n* = 1, control group): participant wore the Manumeter on the unimpaired hand

### 4.1. Wear Time and Effect of Feedback on Hand Activity

**Figure 5:**
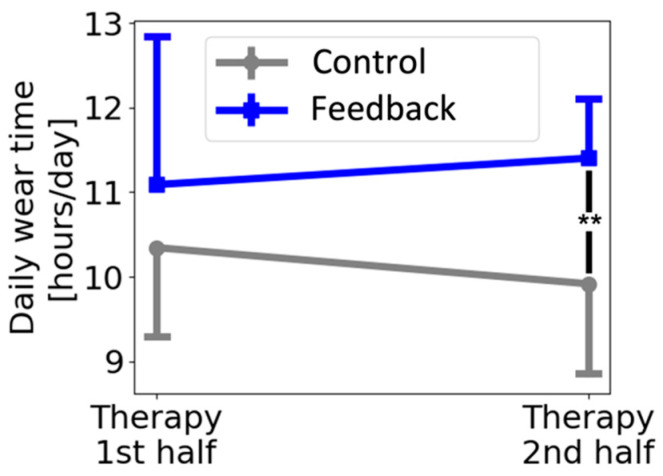
Average daily wear time of the Manumeter. There was a significant difference between groups (*t*-test, *p* = 0.002) during the second half of the three weeks of therapy. Error bars show ±1 standard deviation.

Participants in the feedback and control group wore the Manumeter for 17.2 ± 4.4 and 16.1 ± 3.9 days, respectively—a nonsignificant difference (*t*-test, *p* = 0.66). However, the feedback group wore the Manumeter significantly longer each day compared to the control group, with averages of 11.2 ± 1.3 and 10.1 ± 1.1 h/day (*t*-test, *p* = 0.005), respectively. Comparing the wear time for the two halves of the therapy period (Figure 5), we found no difference between groups for the first half (*t*-test, *p* = 0.238) but a significant difference for the second half (*t*-test, *p* = 0.002).

**Figure 6:**
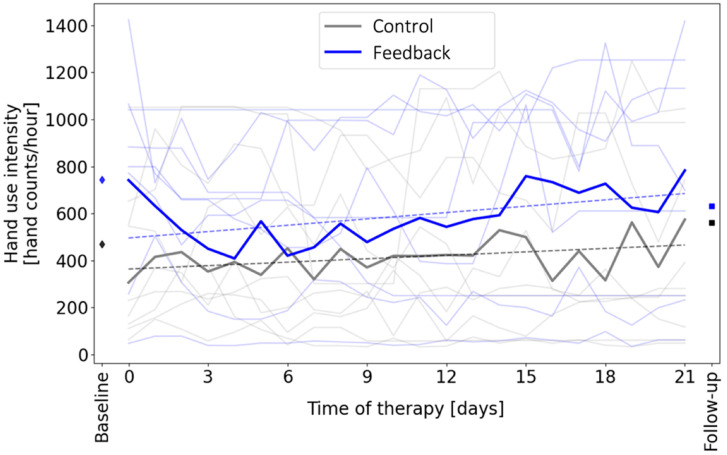
Effect of feedback on hand use intensity. The light lines show data from individuals in order to show variability within and across participants. The dark lines show the group averages. The feedback group significantly increased their hand count intensity over time (dashed line, linear regression, *p* = 0.012, slope = 9.0 hand counts/hour per day, which amounted to ~2000 additional counts per day by study end), while the control group did not (*p* = 0.059; slope = 4.87 hand counts/hour per day). The diamonds and squares show the hand use intensity measured at baseline and follow-up, respectively, when the feedback screen was turned off for all participants. Hand use intensity was not significantly different between groups on the baseline or follow-up days (*t*-test, *p* > 0.05).

The feedback group significantly increased their hand count intensity over time during the intervention period when they received feedback (Figure 6, *p* = 0.012). The control group did not significantly increase hand count intensity over time, although they exhibited an increase that neared significance (*p* = 0.059) (Figure 6). The slopes of the two regression lines shown in Figure 6 were not significantly different (*p* = 0.31). Hand use intensity was not significantly different between groups on the baseline (*p* = 0.22) or follow-up days (*p* = 0.39), when feedback was removed for all participants. Participants achieved their customized daily goal for hand counts on an average of 35% of the 21 days (i.e., on a mean of 7.5 days +/− 7.5 days SD).

### 4.2. Clinical Outcomes

**Figure 7:**
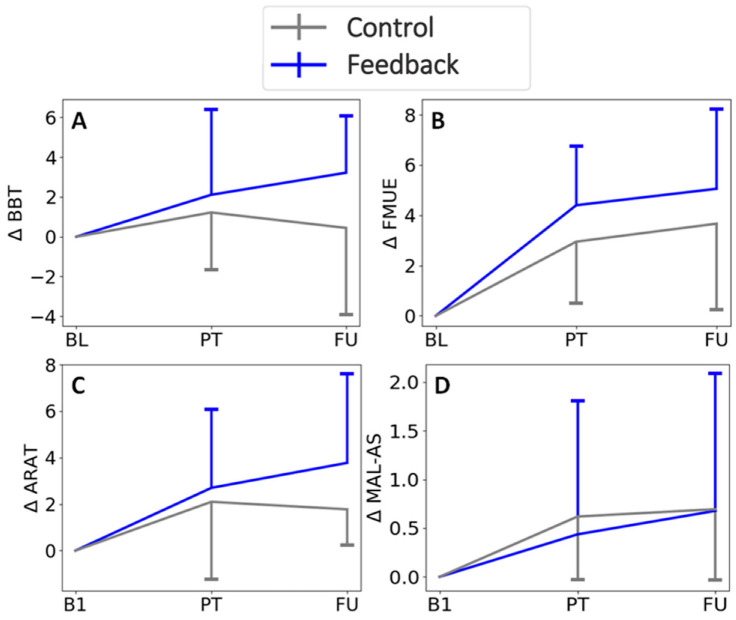
Change in clinical scores from baseline. For BBT and FMUE, B1 is the average for the scores from the two baseline assessments. Bars represent 1 standard deviation. Time points are BL = baseline, B1 = baseline 1, PT = post-therapy, FU = Follow-up. Clinical assessments are BBT = Box and Blocks Test, FMUE = Fugl-Meyer Upper Extremity Scale, ARAT = Action Research Arm Test, MAL-AS = Motor Activity Log—Amount Scale. GroupXtime interactions were not significant.

There was no significant effect of group, and no significant groupXtime interaction, for any of the clinical outcomes (Table 2, Figure 6). There was, however, a significant effect of time on the FMUE, ARAT, and MAL HW, each of which improved over time (Table 2, Figure 7).

### 4.3. Relationship between Hand Performance and Use

**Figure 8:**
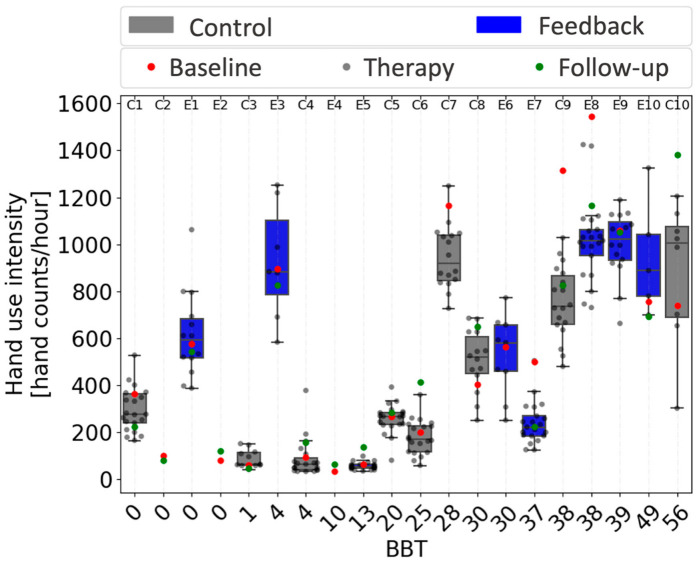
Relationship between hand use intensity and Box and Block Test (BBT) score. Each circle represents the average hourly count for one day. Participants are ordered by BBT. The box and whisker plots use bars to show the 25% and 75% percentiles of daily hand use intensity, and lines to show these quartiles plus and minus the interquartile range. Thus, the plots indicate the individual distribution of daily hand use intensity across the three weeks of therapy for participants in both groups (control in gray, feedback in blue). Box and whisker plots do not include baseline and follow-up data, which are indicated by red and green dots, respectively. Subject numbers are presented on the top, with Es for participants in the feedback group and Cs for participants in the control group. Note that the *x*-axis is equally spaced and not scaled to BBT score.

Figure 8 uses box and whisker plots to summarize the distribution of daily hand use intensity measurements for each participant, as a function of their BBT score. There was a nonlinear relationship between BBT score and hand use intensity, with hand use intensity rising from around the nominal, false positive rate mentioned above (~100–200 counts/hour) only after BBT score exceeded 28, with two exceptions: participants E1 and E3 had low BBT scores but relatively high hand use. We note that participants E1 and E3 were significantly younger (18 and 29 years old) than the remaining participants (averaging 60 ± 10 years old). In addition, compared to all other people with low hand function (BBT < 10, mean FMUE = 26.4 +/− 6.1 SD), these two participants had statistically greater FMUE scores (FMUE = 38 and 31, *p* < 0.05). That is, they were not only younger, but they also had more arm movement ability compared to the other individuals with low hand function.

### 4.4. Possible Baseline Effect

The use of box and whisker plots in Figure 8 makes it clear that three participants (E7, C9, and E8) had an unusually high level of hand use intensity during the baseline monitoring period compared to their other monitoring days. This is because the red dot indicating the baseline value was an outlier, outside of the Q3 + 1.5 *IQR interval for the rest of the acquired data. This suggests that the experience of first wearing the Manumeter produced temporarily greater hand use behavior for these participants. We thus compared baseline hand use intensity with daily hand use intensity, averaged across the three-week study using a one sample *t*-test for all participants. We observed a significant difference for 6 out of 17 subjects (4 greater activity during baseline, 2 lower activity). If this difference were simply random, the odds that it would occur for 6 out of 17 subjects is at most ~1 × 10^−8^ (0.05 to the 6th power). This suggests that the experience associated with a baseline monitoring period changed hand use behavior for a substantial fraction of participants, an observation we discuss further below.

### 4.5. Minimal Detectable Change

**Figure 9:**
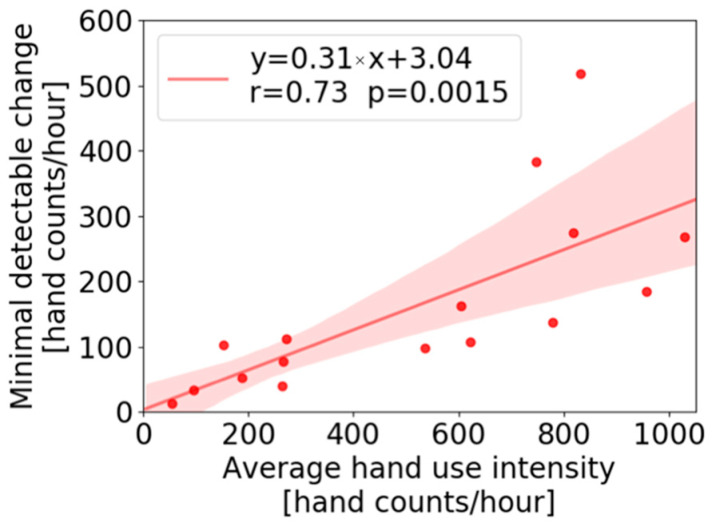
Minimal detectable change in hand use intensity that can be detected with respect to average daily intensity over three weeks of wearing, at a significance level of 0.05, plotted versus average use intensity across the three weeks of wearing. The shaded area represents the 95% confidence interval of the regression line.

The data set we acquired allowed us to calculate the minimal detectable change (MDC) in daily hand use intensity, i.e., how much would hand use intensity have to increase on a given day, relative to its distribution across days of data acquired in the study, in order to be statistically significant? To address this, we calculated the MDC using a one sample *t*-test with a significance level set to 0.05 for each participant, finding the change in magnitude that would be deemed statistically different from the daily hand use intensity values acquired for each participant across the three weeks (Figure 9). A strong, significant correlation (r = 0.73, *p* = 0.0015) was found between the MDC and the average of daily hand use intensity. The slope of this line indicates that a 31% change from the average daily hand use intensity is necessary to detect the change at the 0.05 significance level.

## 5. Discussion

The primary goal of this study was to quantify the effect of real-time wearable feedback of hand counts (accompanied by specification of an individualized daily goal and a conventional home exercise program) on increasing hand activity in chronic stroke. We found that feedback significantly increased hand use intensity over time while it was provided, but did not result in a significant increase in hand use intensity, relative to the control group, that persisted on the follow-up day when the feedback was removed. The participants receiving feedback also wore the Manumeter significantly longer, by about an hour, compared to the no-feedback group, suggesting they were interested in the feedback. There were no significant differences between groups for any clinical measures of hand movement ability that we measured before and after the feedback period, although several of these measures (FMUE, ARAT, and MAL-HW) improved modestly over time. Finally, we confirmed that the previously reported threshold relationship between hand functional capacity and daily use was stable over three weeks, even in the presence of feedback, and we established the MDC for hand count intensity. These results suggest that disuse of the hand after stroke is temporarily modifiable with wearable feedback, but do not support a therapeutic benefit of a brief period of wearable feedback. We first briefly discuss these results, and then limitations and directions for future research.

### 5.1. Resistance to Wearable Feedback after Stroke

We measured a modest increase in hand count intensity of about 20% over time in the feedback group, on average (based on the regression line in Figure 3); this increase occurred gradually over the three weeks in the study. We note that the control group also increased hand count intensity over time at a slower rate, and this change approached significance. Perhaps their increase was attributable to the home exercise program they received; if so, part of the increase in the feedback group may also have been due to engagement in the home exercise program rather than the wearable feedback. The fact that the feedback group wore the Manumeter significantly longer each day does, however, suggest an interest in, and awareness of, the wearable feedback. Regardless of its source, the observed increase in hand count intensity over the time interval examined was modest and did not result in a significant benefit in clinically measured hand functional ability.

It is instructive to compare our results with results from applying wearable feedback to walking activity after stroke. A 2018 Cochrane review examined the evidence regarding the effectiveness of wearable sensors (such as pedometers and smartphone activity monitors) for increasing physical activity levels for people with stroke, finding four studies that met its criteria [21]. These studies compared use of a wearable sensor plus another rehabilitative walking intervention versus the walking intervention alone. The review found no clear effect of the use of wearable sensors on daily step count.

Subsequent studies published since this review have also shown mixed results. For example, Mandigout et al. [22] randomly assigned 83 subacute stroke participants to receive individualized coaching or standard care for six months. The coaches monitored physical activity with an activity tracker, conducted home visits, and made a weekly phone call to review activity. The difference between the two groups was again not significant at any evaluation time point for the primary endpoint, the Six Minute Walk Test. On the other hand, Grau-Pellicer et al. [23] randomized 41 chronic stroke survivors to a conventional rehabilitation program or to a multimodal rehabilitation program that monitored adherence to physical activity. The multimodal program combined an app with GPS and accelerometer-based sensing to monitor walking distance and speed; a pedometer; a WhatsApp group; an exercise program with aerobic, task-oriented, balance, and stretching components; and a progressive daily ambulation program that was monitored by the app and pedometer. At the end of the intervention, community ambulation increased more in the intervention group (38.95 min vs. 9.47 min) and sitting time decreased more in the intervention group (by 3 h/day vs. 0.5 h per day). Thus, in terms of the effectiveness of wearable feedback for improving walking activity after stroke, the results continue to be mixed. However, the Grau-Pellicer et al. study suggests that programs that incorporate wearable sensors into multimodal therapy programs may be more effective than programs that focus solely on goal setting with wearable feedback alone after stroke.

If anything, one might expect even more resistance to increasing hand counts than step counts after stroke. Consider a person who is averaging 400 hand counts per hour, which corresponds roughly to a BBT score of 30. Based on our MDC calculation (Figure 9), this person needs to achieve 120 more hand movements per hour to have a statistically significant increase from baseline. In the context of step counts, taking 120 more steps in a single bout is fairly easy to achieve for unimpaired and even moderately impaired people—it requires 1–2 min of moderate intensity walking [24]. In contrast, in the context of hand counts, it requires a bout of opening and closing the hand 120 times. If the reader tries this, the sense of fatigue will become palpable midway through, even if the reader has no hand impairment. This relative sense of fatigue for generating hand counts versus step counts is attributable to the smaller, more fatigable nature of hand extensor muscles compared to the major muscles of the legs. For people with stroke, this inherent extensor muscle fatiguability compounds with extensor muscle paresis, which is one of the most common consequences of stroke [25]. Perhaps it is then the resulting heightened sense of effort of hand movement after stroke that contributes to hand disuse and makes it resistant to feedback or persisting change. This is consistent with our finding that participants who scored up to ~25 on the BBT rarely used their hand at home, even with feedback. That is, even having a somewhat dexterous, functional hand is not enough to cause regular use of that hand, and we hypothesize that this is because of the increased effort cost associated with using that hand. A possible further confounding factor is the day to day noise in hand counts, which is partly due to measurement noise and partly due to actual variability in daily hand use. This noise caused the MDC to be 30% of the average daily hand counts, which, again, is a difficult target to hit.

If so, expecting wearable feedback to change hand use and improve hand function after stroke without providing intense coaching (or, perhaps, highly motivating gaming environments [26,27]) may be unrealistic. That is, a feedback screen on a watch may be insufficiently motivating in this context. Indeed, participants met daily goals only on about 35% of the days they wore the device. Thus, the number of extra movements achieved may have been too small and/or the types of movements practiced may not have been sufficient to produce a strong therapeutic effect. In contrast, the canonical example of an intense, coached, movement-diverse rehabilitation program is Constraint-Induced Movement Therapy (CIMT), which is typically applied with therapist supervision over several hours per day. The EXCITE trial of CIMT still stands as one of the few successful large-scale trials of UE stroke rehabilitation [28].

### 5.2. Limitations and Future Directions

This study had several limitations. The sample size was small, and the participants had a wide range of impairment levels. It may be that certain subgroups of participants will respond better to wearable feedback, an important topic for future research. Relatedly, we recruited two relatively younger participants. These two younger participants (both in the feedback group and both enrolled in college) had unusually high hand use intensity counts for their impairment level, and unusually low arm impairment for their level of hand function. This suggests that age, occupation, or relative impairment of the proximal versus distal upper extremity can affect hand counts and may need to be controlled for in future studies of wearable feedback.

Another limitation was our use of a single baseline measurement of hand use intensity, which resulted in baseline measurements that were inconsistent with what we observed during the three weeks of intervention. Using as baseline the hours immediately after a visit to our laboratory produced an unusual number of outlier measurements–6 out of 17 participants–in which 4 of them had unusually high hand activity levels, but 2 had unusually low hand activity levels. We hypothesize that these anomalies can be attributed to the disruption of daily routine needed to enroll in the study, and/or motivational effects from meeting with the study team or donning a sensor. For accelerometry, it has been recommended to use at least 3 days of monitoring to estimate habitual physical activity [29].

The Manumeter is sensitive to moderate to large, dynamic changes in hand or wrist position [16]. If the changes were small, or more static/postural rather than dynamic, the Manumeter may not have counted these changes, underestimating increases in hand use intensity.

Another potential limitation was the goal setting strategy. We chose a goal function based on the idea of scaling the goal to BBT ratio, but the assigned goal may not have been optimal for each participant. A possible solution is to use an adaptive goal that encourages a user to move more than their baseline, updated based on average hand use measured across several days.

Although we did not change the goal setting strategy during the study, we changed one component of the feedback—the emoji feedback. This may have produced variability in the results.

A final potential limitation is the form of feedback we used. We relied on participants to visually check their Manumeter displays without providing reminders. There is some indication that providing movement reminders through tactile (i.e., vibratory) inputs to the wrist can increase the amount of UE activity, and that this increase may have at least a small therapeutic benefit [12,30,31,32]. Adding voice capability to the watch could also potentially improve the saliency of the feedback and could be especially valuable for persons with visual impairments.

For future research, we suggest that optimizing the programmatic context in which wearable feedback is delivered will be important for realizing the potential of this technology. It may be necessary to provide wearable feedback over a longer time window, and/or to customize the time window based on response. Other key factors to consider are the way goals are set, the specific form of the performance feedback, the incorporation of reminders, the availability and nature of therapist coaching, and the integration of diverse therapeutic activities along with the wearable feedback.

## Figures and Tables

**Table 1:** Demographic data and key baseline clinical outcomes for RCT.

	All (*n* = 22)	Control (*n* = 11)	Feedback (*n* = 11)
**Age**	57 ± 14	58 ± 12	56 ± 17
**Gender (Male [M]/Female [F])**	18 M/4 F	10 M/1 F	8 M/3 F
**Time since stroke** **(months)**	40 ± 33	48 ± 45	32 ± 14
**Side of hemiparesis** **(Right [R]/Left [L])**	12 R/10 L	8 R/3 L	9 R/2 L
**Type of stroke** **(Ischemic [I]/Hemorrhagic [H])**	12 I/10 H	6 I/5 H	6 I/5 H
**BBT**	20 ± 17	18 ± 18	22 ± 17
**FMUE**	40 ± 13	41 ± 16	40 ± 10
**ARAT**	34 ± 20	34 ± 21	34 ± 18

± standard deviation.

**Table 2:** Changes in clinical score in the RCT. Delta PT = change from baseline to post-therapy evaluation, Delta FU = change from baseline to follow-up evaluation.

	Significance (*p* Value)			Delta PT		Delta FU	
Outcome	Group	Time	GroupXTime	Control	Feedback	Control	Feedback
**BBT**	0.608	0.116	0.353	1.2 ± 2.7	2 ± 4.1	0.44 ± 4.4	3.2 ± 2.8
**FMUE**	0.94	<0.001 ***	0.454	3 ± 2.5	4.4 ± 2.3	3.7 ± 3.5	5.1 ± 3.2
**ARAT**	0.83	0.002 **	0.416	2.1 ± 3.4	2.7 ± 3.3	1.8 ± 1.5	3.8 ± 3.8
**NIHSS**	0.259	0.555	0.564	−0.2 ± 0.98	−0.1 ± 0.54	0 ± 0.94	−0.33 ± 0.47
**Gross grasp [kg]**	0.717	0.654	0.629	−1.3 ± 2	−0.4 ± 0.92	−1.1 ± 1.7	−0.78 ± 1.5
**Lateral pinch [kg]**	0.275	0.616	0.334	−1.1 ± 1.8	−0.35 ± 1.8	−0.89 ± 1.4	−0.61 ± 2.4
**MAL AS**	0.969	0.065	0.917	0.62 ± 0.66	0.44 ± 1.4	0.69 ± 0.73	0.68 ± 1.4
**MAL HW**	0.884	0.017 *	0.788	0.56 ± 0.75	0.35 ± 0.52	0.58 ± 0.8	0.44 ± 0.7
**MMSE**	0.065	0.662	0.567	0.4 ± 1.7	−0.2 ± 1.1	−0.11 ± 0.74	−0.33 ± 0.47

N = 10 for each group (note: missing 1 participant in each group for FU evaluation). Significance level: * *p* < 0.5; ** *p* < 0.01; *** *p* < 0.001. ± indicates standard deviation.

## Data Availability

The data presented in this study are available on request from the corresponding author. The data are not publicly available due to privacy.
